# Neuromyths in Education: Prevalence and Predictors of Misconceptions among Teachers

**DOI:** 10.3389/fpsyg.2012.00429

**Published:** 2012-10-18

**Authors:** Sanne Dekker, Nikki C. Lee, Paul Howard-Jones, Jelle Jolles

**Affiliations:** ^1^Department of Educational Neuroscience, Faculty of Psychology and Education, LEARN! Institute, VU University AmsterdamAmsterdam, Netherlands; ^2^Graduate School of Education, University of BristolBristol, UK

**Keywords:** neuromyths, educational neuroscience, prevalence, predictors, teachers

## Abstract

The OECD’s Brain and Learning project (2002) emphasized that many misconceptions about the brain exist among professionals in the field of education. Though these so-called “neuromyths” are loosely based on scientific facts, they may have adverse effects on educational practice. The present study investigated the prevalence and predictors of neuromyths among teachers in selected regions in the United Kingdom and the Netherlands. A large observational survey design was used to assess general knowledge of the brain and neuromyths. The sample comprised 242 primary and secondary school teachers who were interested in the neuroscience of learning. It would be of concern if neuromyths were found in this sample, as these teachers may want to use these incorrect interpretations of neuroscience findings in their teaching practice. Participants completed an online survey containing 32 statements about the brain and its influence on learning, of which 15 were neuromyths. Additional data was collected regarding background variables (e.g., age, sex, school type). Results showed that on average, teachers believed 49% of the neuromyths, particularly myths related to commercialized educational programs. Around 70% of the general knowledge statements were answered correctly. Teachers who read popular science magazines achieved higher scores on general knowledge questions. More general knowledge also predicted an increased belief in neuromyths. These findings suggest that teachers who are enthusiastic about the possible application of neuroscience findings in the classroom find it difficult to distinguish pseudoscience from scientific facts. Possessing greater general knowledge about the brain does not appear to protect teachers from believing in neuromyths. This demonstrates the need for enhanced interdisciplinary communication to reduce such misunderstandings in the future and establish a successful collaboration between neuroscience and education.

## Introduction

There is widespread interest among teachers in the application of neuroscientific research findings in educational practice. Neuroscientific research has received a lot of attention since 1990–2000, which was declared the “Decade of the Brain” in the United States. Yet, the field of neuroscience is complex and the accurate transfer of research findings to the classroom is often difficult (Jolles et al., 2005; Devonshire and Dommett, 2010; Ansari et al., 2011). This gap between neuroscience and education has enabled many misconceptions about scientific findings to occur (Goswami, 2006). In 2002, the Brain and Learning project of the Organization for Economic Co-operation and Development (OECD) drew international attention to this phenomenon. The organization raised concerns with regards to the rapid proliferation of so-called “neuromyths”. These were defined as “a misconception generated by a misunderstanding, a misreading, or a misquoting of facts scientifically established (by brain research) to make a case for use of brain research in education and other contexts” (Organisation for Economic Co-operation, and Development, 2002). The influence of these myths in the classroom is problematic because it wastes money, time, and effort, which could be better spent on the development of evidence-based practices (Sylvan and Christodoulou, 2010; Pasquinelli, 2012). Despite concerns regarding the rapid proliferation of neuromyths (e.g., Goswami, 2006), not much is known about the prevalence of neuromyths among professionals in the field of education. The current study investigated if belief in neuromyths was common among teachers that were interested in the neuroscience of learning. It would be of concern if neuromyths were found in this sample, because these teachers will be most eager to implement (wrong) brain-based ideas in educational practice. Furthermore, these teachers might promote the circulation of myths and spread their ideas to teachers who are less engaged and acknowledged with brain research. In addition to examining the prevalence of neuromyths, the study also investigated which myths were most and least prevalent. To shed light on how the proliferation of myths may differ between countries, teachers from specific regions in both the United Kingdom (UK) and the Netherlands (NL) were involved. Additionally, this study focused on identifying factors that predict belief in neuromyths.

Although neuromyths are incorrect assertions about how the brain is involved in learning, their origin often lies in genuine scientific findings. An example of a neuromyth is that learning could be improved if children were classified and taught according to their preferred learning style. This misconception is based on a valid research finding, namely that visual, auditory, and kinesthetic information is processed in different parts of the brain. However, these separate structures in the brain are highly interconnected and there is profound cross-modal activation and transfer of information between sensory modalities (Gilmore et al., 2007). Thus, it is incorrect to assume that only one sensory modality is involved with information processing. Furthermore, although individuals may have preferences for the modality through which they receive information [either visual, auditory, or kinesthetic (VAK)], research has shown that children do not process information more effectively when they are educated according to their preferred learning style (Coffield et al., 2004). Other examples of neuromyths include such ideas as “we only use 10% of our brain”, “there are multiple intelligences”, “there are left- and right brain learners”, “there are critical periods for learning” and “certain types of food can influence brain functioning” (e.g., Organisation for Economic Co-operation, and Development, 2002; Geake, 2008; Purdy, 2008; Howard-Jones, 2010). Some of these misunderstandings have served as a basis for popular educational programs, like Brain Gym or the VAK approach (classifying students according to a VAK learning style). These programs claim to be “brain-based” but lack scientific validation (Krätzig and Arbuthnott, 2006; Waterhouse, 2006; Stephenson, 2009; Lindell and Kidd, 2011). A fast commercialization has led to a spread of these programs into classrooms around the world.

Yet, only a few studies have examined the prevalence of misunderstandings about the mind and brain. A study examining neuroscience knowledge in the general population of Brazil revealed that many misconceptions existed among the general public, and that there was a lot of variation in the frequency of these misunderstandings (Herculano-Houzel, 2002). The statement “we only use 10% of our brain”, defined by the Organisation for Economic Co-operation, and Development (2002) as a neuromyth, was the most prevalent misconception among the public. Neuromyths were also found to be prevalent among trainee teachers (Howard-Jones et al., 2009). In particular, myths related to commercial brain-based educational programs were commonly accepted. Furthermore, the research showed that many trainee teachers in the UK (56–83%) had encountered one or more of these commercial brain-based programs (e.g., Brain Gym or VAK approach) in their school. To our knowledge, no studies have examined the prevalence of neuromyths among teachers who are interested in the neuroscience of learning. Furthermore, it is unclear how the implementation of brain-based programs differs across countries.

Next to examining the prevalence of neuromyths, it is important to identify the factors that predict a high susceptibility to believing in myths. Experimental research has shown that people are generally more likely to believe research findings when they are accompanied by brain images and neuroscience explanations, even when these are incorrect (Weisberg et al., 2007; McCabe and Castel, 2008). Weisberg et al. (2007) found that the public’s perception of a poor explanation became more positive when neuroscience was included, even though the neuroscience was irrelevant. This may lead to misjudgments of scientific evidence. Furthermore, it may be difficult for people who lack neuroscientific expertise to recognize misconceptions about brain research in the popular media. Information provided by the popular media is often over-simplified or over-interpreted, as the popular media aims to reach many people. Therefore, popular media have been held responsible for creating misconceptions (Wallace, 1993; Beck, 2010). Apparent simplicity in popular articles may lead to the flawed assumption that complex neuroscience is easily applicable in the classroom. When people lack a general understanding of the brain and do not critically reflect on their readings, they may be more vulnerable to neuromyths. Thus, a lack of neuroscience literacy and reading popular media may be factors that predict the number of misconceptions teachers have about the brain.

Consequently, neuroscience literacy (i.e., a general understanding of the brain) may protect against incorrect ideas linking neuroscience and education. Support for this hypothesis was found in a sample of trainee teachers (Howard-Jones et al., 2009), where general knowledge of the brain related positively to the ability to identify neuromyths. This suggests that neuroscience literacy is an important factor that enables individuals to differentiate science from pseudoscience. Fortunately, many teachers are eager to increase their neuroscience literacy (Pickering and Howard-Jones, 2007; Hook and Farah, 2012). Attempts to increase their literacy most often included in-service training about the brain (Pickering and Howard-Jones, 2007), which has been identified as a strong predictor of neuroscience literacy (Herculano-Houzel, 2002). Next to in-service training, neuroscience literacy has been predicted by reading popular science magazines and newspapers (Herculano-Houzel, 2002). Thus, reading popular media seems to have both beneficial effects (higher neuroscience literacy) and negative effects (creating misconceptions).

The present study investigated the neuroscience literacy and prevalence of neuromyths among primary and secondary school teachers in the UK and the NL. The sample consisted of teachers who indicated they were interested in the neuroscience of learning. It would be of concern if neuromyths were found in this sample, as these teachers may want to use these incorrect interpretations of neuroscience findings in their teaching practice. By including teachers from both the UK and the NL, it was possible to examine possible differences between countries and the educational systems therein. The study aimed to give an indication of the prevalence and predictors of myths among teachers in primary and secondary school. It is therefore of potential importance for the development of educational innovations which target teachers’ knowledge of neuroscience. The second aim of the study was to examine a range of factors that might be associated with belief in neuromyths such as reading popular science magazines. Teachers completed a survey comprising neuromyths and general assertions about the brain and its involvement in learning. The hypotheses were that myths related to commercialized educational programs would be the most prevalent of the myths presented. General knowledge and in-service training were expected to have a protective effect on the belief in myths. Furthermore, it was hypothesized that teachers who read popular science magazines would believe more neuromyths. Therefore, we additionally investigated whether certain teacher characteristics (e.g., age, sex, primary/secondary school teacher) were associated with knowledge and the number of myths. By this means, the study will provide valuable information about the possible prevention of neuromyths in education.

## Materials and Methods

### Participants

The total sample of 242 participants included 137 teachers from the Dorset region of the UK and 105 teachers from several regions in the NL surrounding the Amsterdam area. Participants were primary school teachers (44%), secondary school teachers (50%), and other teachers (e.g., trainee teachers, teachers in special education, teaching assistants; 6%). The schools from which the teachers were drawn could be considered a random selection of primary and secondary schools in the UK and the NL. Teachers from both countries were comparable in age [*M* age = 43 years, SD = 11.0; *t*(180.3) = 1.16, *p* = 0.249]. Furthermore, the distribution of primary school teachers, secondary school teachers, and other teachers was the same in both countries [χ*^2^*(2, *N* = 241) = 2.42, *p* = 0.298]. The UK sample comprised relatively more female teachers (77%) than the Dutch sample (64%), χ*^2^*(1, *N* = 240) = 5.05, *p* = 0.025. The male/female ratio did not differ between primary school, secondary school, and other teachers [χ*^2^*(2, *N* = 240) = 5.28, *p* = 0.071]. Of all teachers, 93% were interested in scientific knowledge about the brain and its influence on learning. Further, 90% of the teachers thought that this knowledge was very valuable for their teaching practice.

### Procedure

Schools in the selected regions were approached for participation in the research project. They were asked to forward an email with information about the research project to all teachers in their school. The research was presented as a study of how teachers think about the brain and its influence on learning. The term neuromyth was not mentioned in the information for teachers. Teachers who were interested in this topic and chose to participate, followed a link to an online survey. Average completion time was 15 min.

### Measures

The online survey included 32 statements about the brain and its influence on learning (see Appendix). It comprised 15 statements that were educational neuromyths, as defined by the Organisation for Economic Co-operation, and Development (2002) and Howard-Jones et al. (2009), e.g., “Individuals learn better when they receive information in their preferred learning style (e.g., auditory, visual)”. The other 17 statements were general assertions about the brain, e.g., “The left and right hemisphere of the brain always work together”. The presentation order of myth and knowledge assertions was randomized. Answer options were “incorrect”, “correct”, or “do not know”. Correct and incorrect assertions were balanced. Dependent variables were the percentage of incorrect answers on neuromyth assertions (where a higher percentage reflects more belief in myths) and the percentage of correct responses on general assertions.

Additionally, teachers provided background information about their age, sex, level of education [graduate or postgraduate, and whether they had a Postgraduate Certificate in Education (PGCE)] and whether they were a teacher in primary or secondary school. They indicated whether they were interested in scientific knowledge about the brain and its influence on learning and whether they thought this knowledge was very valuable for their teaching practice. Also, they estimated the role of genes and environment in learning. Furthermore, they were asked whether they followed any in-service or other training about the brain and whether they encountered educational approaches that claimed to be brain-based in their school (Brain Gym, Learning styles, Multiple Intelligences, Left/right brain learners). Further, they indicated whether they read popular science magazines and/or scientific journals.

### Data analysis

The data was analyzed using the Statistical Package for the Social Sciences (SPSS) version 17.0 for Windows. For all analysis, a statistical threshold of α = 0.05 was used. Independent *t*-tests were performed to examine differences between countries (independent variable) in percentage of neuromyths and percentage of correct responses on general statements (dependent variables). To examine which factors predicted neuromyths, a regression analysis was performed for percentage of myths (dependent variable) with country, sex, age, school type (primary/secondary school), reading popular science, reading scientific journals, in-service training, and percentage of correct answers on general assertions (predictors). A second regression analysis was performed to examine the predictors of neuroscience literacy. Percentage of correct answers on general assertions was the dependent variable, and predictors were country, sex, age, school type (primary/secondary school), reading popular science, reading scientific journals, and in-service training.

## Results

### Prevalence of neuromyths

Overall, teachers agreed with 49% of the statements promoting myths indicating that they believed these myths. There was no significant difference in overall prevalence between countries [*t*(240) = 0.408, *p* = 0.684]. An analysis of the responses for each myth showed a lot of variation between the myths (see Table 1). Seven of the 15 myth statements were believed by more than 50% of the teachers. The most prevalent of these myths were (1) “Individuals learn better when they receive information in their preferred learning style (e.g., auditory, visual, kinesthetic)”, (2) “Differences in hemispheric dominance (left brain, right brain) can help explain individual differences amongst learners”, and (3) “Short bouts of co-ordination exercises can improve integration of left and right hemispheric brain function”. More than 80% of the teachers believed these myths. Other statements related to neuromyths were often successfully identified, e.g., “Individual learners show preferences for the mode in which they receive information (e.g., visual, auditory, kinesthetic)”. More than 80% of the teachers answered this statement correctly.

**Table 1:** **Correctness of responses for each myth assertion**.

Neuromyth	Incorrect		Correct		Do not know	
	UK (%)	NL (%)	UK (%)	NL (%)	UK (%)	NL (%)
Individuals learn better when they receive information in their preferred learning style (e.g., auditory, visual, kinesthetic).	93	96	4	3	3	1
Differences in hemispheric dominance (left brain, right brain) can help explain individual differences amongst learners.	91	86	3	4	6	11
Short bouts of co-ordination exercises can improve integration of left and right hemispheric brain function.	88	82	0	5	12	13
Exercises that rehearse co-ordination of motor-perception skills can improve literacy skills.	78	63	3	11	19	27
Environments that are rich in stimulus improve the brains of pre-school children.	95	56	1	29	4	15
Children are less attentive after consuming sugary drinks, and/or snacks.	57	55	24	24	20	21
It has been scientifically proven that fatty acid supplements (omega-3 and omega-6) have a positive effect on academic achievement.	69	54	12	16	20	30
There are critical periods in childhood after which certain things can no longer be learned.	33	52	53	38	14	10
We only use 10% of our brain.	48	46	26	42	26	12
Regular drinking of caffeinated drinks reduces alertness.	26	36	39	41	35	23
Children must acquire their native language before a second language is learned. If they do not do so neither language will be fully acquired.	7	36	82	61	11	3
Learning problems associated with developmental differences in brain function cannot be remediated by education.	16	19	69	62	15	19
If pupils do not drink sufficient amounts of water (=6–8 glasses a day) their brains shrink.	29	16	46	49	26	35
Extended rehearsal of some mental processes can change the shape and structure of some parts of the brain.	6	14	69	58	26	28
Individual learners show preferences for the mode in which they receive information (e.g., visual, auditory, kinesthetic).	4	13	95	82	2	5

With respect to the general statements about the brain, a difference between countries was found [*t*(240) = −3.09, *p* = 0.002]: Dutch teachers had higher scores on general knowledge (*M *= 73% correct, SD = 12.7) than teachers from the UK (*M *= 67% correct, SD = 13.5). Scores on knowledge did not vary with the teachers’ level of education [UK: *F*(4, 133) = 0.48, *p* = 0.748; NL: *F*(3, 104) = 0.41, *p* = 0.743]. Furthermore, there were no differences between primary and secondary school teachers [*t*(224) = −0.15, *p* = 0.879].

Brain Gym (Brain Gym International, 2011), Learning Styles, and Left brain/Right brain learning programs were encountered significantly more often in schools in the UK than in the NL (see Table 2). More teachers from the UK than the NL followed in-service training. Dutch teachers read popular science magazines or scientific journals more often than teachers in the UK (see Table 2). There were significant differences between counties in teachers’ views on the role of genes and environment in learning. Teachers in the NL gave considerably greater weight to genes than teachers in the UK (34 vs. 22%). Teachers in the UK attributed more to home environment (46%) and school environment (29%), compared to Dutch teachers (resp. 30 and 25%).

**Table 2:** **Teacher characteristics**.

	UK (%)	NL (%)
Encountered in school		
Brain gym	82	8
Learning styles	98	64
Multiple intelligences	71	67
Left/right brain learners	44	18
Followed in-service training	66	34
Read popular science	28	73
Read scientific journals	38	62

### Predictors of neuromyths and knowledge

Belief in myths was significantly predicted by general knowledge of the brain (β = 0.24; see Table 3). This indicates that teachers with higher scores on knowledge were more likely to believe in myths. None of the other factors [country, sex, age, school type (primary/secondary school), reading popular science, reading scientific journals, or in-service training] predicted belief in myths. The model explained a significant proportion of variance (*R*^2^ = 0.089) in myth scores, *F*(8, 210) = 2.463, *p* = 0.014.

**Table 3:** **Predictors of neuromyths**.

	*B* (SE)	*t*	*p*	95% CI for *B*	
				Lower	Upper
Intercept	0.250 (0.067)	3.73	0.000	0.118	0.382
Country	−0.001 (0.020)	−0.072	0.943	−0.041	0.038
Age	0.002 (0.001)	1.75	0.082	0.000	0.003
Gender	0.030 (0.021)	1.43	0.155	−0.011	0.071
Teacher	−0.024 (0.019)	−1.27	0.206	−0.061	0.013
Read popular science	0.006 (0.024)	0.256	0.798	−0.041	0.053
Read scientific journals	−0.024 (0.026)	−0.940	0.348	−0.075	0.027
In-service training	−0.002 (0.020)	−0.078	0.938	−0.040	0.037
Knowledge (% correct)	0.240 (0.071)	3.39	0.001*	0.100	0.379

*p < 0.001.

General knowledge of the brain was predicted by country (β = 0.16) and reading popular science magazines (β = 0.21; see Table 4). This shows that knowledge was higher among Dutch teachers, and among teachers who read popular science magazines. Age, sex, school type, reading scientific journals, and following in-service training did not relate to scores on knowledge. The model explained 10% of the variance, which was significant, *F*(7, 210) = 3.24, *p* = 0.003.

**Table 4:** **Predictors of general knowledge**.

	*B* (SE)	*t*	*p*	95% CI for *B*	
				Lower	Upper
Intercept	0.678 (0.046)	14.631	0.000	0.587	0.769
Country	0.044 (0.02)	2.270	0.024*	0.006	0.083
Age	−0.001 (0.001)	−0.688	0.492	−0.002	0.001
Gender	−0.005 (0.021)	−0.238	0.812	−0.046	0.036
Teacher	−0.002 (0.019)	−0.122	0.903	−0.039	0.034
Read popular science	0.067 (0.023)	2.919	0.004**	0.022	0.113
Read scientific journals	0.002 (0.026)	0.065	0.948	−0.049	0.052
In-service training	0.035 (0.019)	1.814	0.071	−0.003	0.073

*p < 0.05; **p < 0.01.

## Discussion

This study examined general knowledge about the brain and prevalence of neuromyths among teachers in specific regions of the UK and the NL. It additionally investigated a range of candidate factors that might be associated with these outcomes. The results indicated that, overall, teachers believed half of the presented myths. Seven of the 15 myths were believed by more than half of the teachers. The most prevalent myths related to Brain Gym (Brain Gym International, 2011), learning styles, and left brain/right brain learners. The prevalence of the different myths varied between countries. A higher incidence of myths (higher percentage of questions answered incorrectly) was predicted by higher general knowledge of the brain. The average score on general knowledge of the brain was around 70%. A higher number of correct answers on general statements was predicted by reading popular science magazines. Furthermore, general knowledge about the brain was higher among Dutch teachers. Teacher characteristics (age, sex, primary/secondary school teacher) did not predict literacy or belief in neuromyths.

These results validate previously voiced concerns about the proliferation of neuromyths in the field of education (Organisation for Economic Co-operation, and Development, 2002; Goswami, 2006). They emphasize that teachers who are highly interested in brain research are susceptible to neuromyths. This is troublesome, as these teachers in particular may implement wrong brain-based ideas in educational practice. Misconceptions related to brain-based educational programs were most prevalent, as was also found in a previous study with trainee teachers (Howard-Jones et al., 2009). This suggests that these programs have been successfully marketed within schools ever since the “Decade of the Brain”. The prevalence of misconceptions was found to vary across countries. This might be due to differences across countries regarding the marketing of brain-based programs (Pasquinelli, 2012). For instance, in the NL, there is less marketing of these brain-based programs. There were no differences between countries in terms of general knowledge about the brain. This suggests similar familiarity with brain research between teachers from both countries.

The present research showed that knowledge about the brain was higher when teachers read popular science magazines. Teachers who are eager to learn about the brain and its possible applications in the classroom may more often search for information in the popular media. Furthermore, teachers’ views on the role of genetics and environment on learning was investigated. In our survey, teachers in both the UK and NL gave considerably greater weight to the environment, with UK teachers attributing only 22% to genetic factors. This is close to the figure of 25% amongst UK trainee teachers surveyed by Howard-Jones et al. (2009). Yet, Walker and Plomin (2005) also surveyed UK teachers and concluded that the perception of their teachers that genetics was at least as important as environment in most areas was in line with research indicating substantial genetic influence on these domains (e.g., Plomin et al., 2001). Differences between the studies might be related to confusion over the term “environment”, as suggested by Howard-Jones et al. (2009). This term can have a range of disparate meanings in education, most of which are narrower than its meaning within the field of genetics, and many of which may not even include the teacher’s efforts.

In contrast to our hypothesis and earlier findings by Howard-Jones et al. (2009) in a sample of trainee teachers, we did not find a protective effect of knowledge on belief in myths. Instead, our results showed that belief in neuromyths correlated positively with general knowledge about the brain. It may be that a lot of interest in the brain has served trainees well in developing their general awareness about the brain. However, teachers who have worked in the field of education for a number of years, will have been confronted with more information about the brain and its influence on learning, both correct and incorrect. Apparently, it is difficult for teachers to then differentiate between this correct and incorrect information. This might be attributed to their eagerness to implement knowledge about the brain in educational practice, in combination with a lack of expertise in neuroscience. Although some of the teachers in our sample followed in-service training about the brain, none of them were experts in the field of neuroscience. Experiments by Weisberg have shown that people with some neuroscientific knowledge (people who followed an introductory cognitive neuroscience class) were fooled by neuroscientific explanations in the same way as laypeople. Only neuroscience experts (defined as people who were about to pursue or had a degree in cognitive neuroscience or related areas) were able to correctly identify non-sense neuroscientific findings. Thus, the level of knowledge of teachers in our sample was not sufficient to protect them against the general credibility of neuroscience findings. When teachers are eager to implement neuroscientific findings, but lack expertise in neuroscience and seek quick and easy solutions, they may fail to recognize misconceptions.

Besides the fact that it wastes money, time, and effort, the implementation of myths in the classroom should be prevented because it may diminish teachers’ confidence in a successful collaboration between the fields of neuroscience and education (Sylvan and Christodoulou, 2010; Pasquinelli, 2012). To reduce the number of myths that currently proliferate within schools, we would welcome explicit education for teachers about neuromyths and the lack of scientific evidence for many “brain-based” programs. Previous research has shown that this can be effective in reducing the incidence of misconceptions (Kowalski and Taylor, 2009; Dommett et al., 2011).

To avoid the occurrence of misconceptions in the future, we suggest improving the communication between scientists and practitioners, in addition to enhancing the neuroscience literacy of teachers. Incorporating neuroscience courses into initial teacher training could enhance neuroscience literacy among teachers. In addition, initial teacher training should include the skills needed to evaluate scientific research (Lilienfeld et al., 2012). This would enable teachers to develop a critical attitude toward the information they receive and examine scientific evidence before including neuroscientific findings into their teaching practice (Howard-Jones, 2009). At the same time, scientists are advised to check translations of their research for the popular media carefully. They should clearly explain what can and what cannot be concluded from their data (Beck, 2010). As some familiarity with brain research was not enough to distinguish myths from the truth, the present study highlights the importance of a dialog between teachers and neuroscience experts in order to establish effective collaborations between the two fields (Jolles et al., 2005; Hruby, 2012). As Dommett et al. (2011) showed, a possible framework for how this could be achieved is to let teachers decide on the topics of neuroscience workshops and to spend considerable time on dialog between neuroscientists and teachers to reflect on the translation of this knowledge to classroom practices.

The present results reflect the prevalence of neuromyths in a sample of teachers with a strong interest in the neuroscience of learning. This yields important information about teachers who may implement wrong brain-based ideas in educational practice. However, average scores on general knowledge and myth assertions may be somewhat different in the population of teachers as a whole. Teachers who are less interested in brain research may believe even more myths, due to a lack of knowledge about neuroscience and a lack of motivation to unravel difficult findings from brain research. For future research, it is important to examine where teachers’ incorrect ideas originate (e.g., books, colleagues, commercial companies) and to perform intervention studies directed at increasing teacher competence in understanding the functioning of the brain. Such intervention studies should be performed according to the principles and approach of evidence-based or evidence-informed practice. This could yield valuable information for the prevention of myths in the future and for the development of valid educational innovations.

In conclusion, this research suggests that teachers who are enthusiastic about the possible application of neuroscience findings in the classroom, often find it challenging to distinguish pseudoscience from scientific facts. Possessing greater general knowledge about the brain does not appear to protect teachers from picking up neuromyths. This demonstrates the need to enhance teacher professionalism and interdisciplinary communication to reduce such misunderstandings in the future. It is encouraging that teachers are eager to learn about the brain and its role in learning. Although the integration of neuroscience in educational practice remains challenging, joint efforts of scientists and practitioners may pave the way toward a successful collaboration between the two fields.

## Conflict of Interest Statement

The authors declare that the research was conducted in the absence of any commercial or financial relationships that could be construed as a potential conflict of interest.

## Appendix

1. We use our brains 24 h a day (C).
2. *Children must acquire their native language before a second language is learned. If they do not do so neither language will be fully acquired (I)*.
3. Boys have bigger brains than girls (C).
4. *If pupils do not drink sufficient amounts of water (*=*6–8 glasses a day) their brains shrink (I)*.
5. *It has been scientifically proven that fatty acid supplements (omega-3 and omega-6) have a positive effect on academic achievement (I)*.
6. When a brain region is damaged other parts of the brain can take up its function (C).
7. *We only use 10% of our brain (I)*.
8. The left and right hemisphere of the brain always work together (C).
9. *Differences in hemispheric dominance (left brain, right brain) can help explain individual differences amongst learners (I)*.
10. The brains of boys and girls develop at the same rate (I).
11. Brain development has finished by the time children reach secondary school (I).
12. *There are critical periods in childhood after which certain things can no longer be learned (I)*.
13. Information is stored in the brain in a network of cells distributed throughout the brain.
14. Learning is not due to the addition of new cells to the brain (C).
15. *Individuals learn better when they receive information in their preferred learning style (e.g*., *auditory*, *visual*, *kinesthetic) (I)*.
16. Learning occurs through modification of the brains’ neural connections (C).
17. Academic achievement can be affected by skipping breakfast (C).
18. Normal development of the human brain involves the birth and death of brain cells (C).
19. Mental capacity is hereditary and cannot be changed by the environment or experience (I).
20. Vigorous exercise can improve mental function (C).
21. *Environments that are rich in stimulus improve the brains of pre-school children (I)*.
22. *Children are less attentive after consuming sugary drinks and/or snacks (I)*.
23. Circadian rhythms (“body-clock”) shift during adolescence, causing pupils to be tired during the first lessons of the school day (C).
24. *Regular drinking of caffeinated drinks reduces alertness (C)*.
25. *Exercises that rehearse co-ordination of motor-perception skills can improve literacy skills (I)*.
26. *Extended rehearsal of some mental processes can change the shape and structure of some parts of the brain (C)*.
27. *Individual learners show preferences for the mode in which they receive information (e.g*., *visual*, *auditory*, *kinesthetic) (C)*.
28. *Learning problems associated with developmental differences in brain function cannot be remediated by education (I)*.
29. Production of new connections in the brain can continue into old age (C).
30. *Short bouts of co-ordination exercises can improve integration of left and right hemispheric brain function (I)*.
31. There are sensitive periods in childhood when it’s easier to learn things (C).
32. When we sleep, the brain shuts down (I).
33. Neuromyth assertions are presented in *italic*; C = correct; I = incorrect.
